# Differences in Action Style Recognition in Children with Autism Spectrum Disorders

**DOI:** 10.3389/fpsyg.2017.01456

**Published:** 2017-09-04

**Authors:** Giuseppe Di Cesare, Laura Sparaci, Annalisa Pelosi, Luigi Mazzone, Giulia Giovagnoli, Deny Menghini, Emanuele Ruffaldi, Stefano Vicari

**Affiliations:** ^1^Department of Robotics, Brain and Cognitive Sciences, Istituto Italiano di Tecnologia Genoa, Italy; ^2^Neuroscience Unit, Department of Medicine and Surgery, University of Parma Parma, Italy; ^3^Institute of Cognitive Sciences and Technologies, National Research Council Rome, Italy; ^4^Experimental Psychology, University College London London, United Kingdom; ^5^IRCCS Children’s Hospital Bambino Gesù Rome, Italy; ^6^Child Neuropsychiatry Unit, Department of Neuroscience, IRCCS Children’s Hospital Bambino Gesù Rome, Italy; ^7^Laboratory of Perceptual Robotics, Institute of Communication, Information and Perception Technologies, Scuola Superiore Sant’Anna Pisa, Italy

**Keywords:** autism, social behavior, action observation, action style, vitality forms

## Abstract

Vitality form is a term, originally introduced by Stern (2010), to describe “how” an action is performed. The capacity to perceive the vitality form of others’ actions is a fundamental element of social interactions and a basic way of relating to and understanding others’ behaviors. Although vitality forms characterize all human interactions, few studies have addressed their role in social and communicative disorders such as autism. The aim of the present study is to evaluate the ability to recognize different vitality forms during the observation of different motor actions in a group of children with autism spectrum disorders (ASD) compared to typically developing controls (TD). Results show a significant difference between children with ASD and TD in vitality forms recognition. This finding sheds new light on how children with ASD understand others’ actions providing new ideas on overall social understanding as well as useful insights for professionals and caregivers alike.

## Introduction

Important information about others’ behavior is conveyed by the dynamics of observed actions’ style, which have been named “vitality forms” by Stern (2010). Action style or vitality form represent “how” an action is performed, characterizing an important aspect that an observer may capture when he views an action performed by others. Vitality forms must be kept distinct from “what” action is being performed and “why” it is being performed. For example, if someone grasps a mug to drink, the goal-directed grasp is the content of the movement (i.e., “what”), while drinking is the goal or motor intention (i.e., “why”), but if the mug is grasped vigorously or gently, is the manner or style in which the action is executed (i.e., the “how” or vitality form). This distinction is not merely conceptual, but also anatomical, as different neural structures have been shown to become active during “what” and “how” judgments. For example, in an fMRI study by Di Cesare et al. (2013) a group of healthy adults was asked to observe two actors performing actions with objects (e.g., giving a mug) with different vitality forms (i.e., energetic or gentle) and to focus either on “what” action was performed or on “how” it was being performed. Results showed that although “what” and “how” tasks produced activation of similar brain regions, such as occipito-temporal areas and the fronto-parietal circuit, the “how” task showed enhanced activation in the right dorso-central insular cortex. While this study focused on visual information, it is important to stress that vitality forms may be conveyed by both visual and auditory information. In a recent study Di Cesare et al. (2017) demonstrated that the central insula is a key region involved in both acoustical and visual vitality form processing. Furthermore, vitality forms do not coincide with emotions, since a single emotion (e.g., anger), may be expressed through multiple vitality forms (e.g., it may “explode,” “ooze out,” or “sneak up”; Stern, 2010). In fact, the perception of vitality forms does not lead to increased activation in the anterior insula, which is usually involved in processing emotional states like anger and fear (e.g., Wicker et al., 2003; Gallese et al., 2004; Singer et al., 2004; Grosbras and Paus, 2005; de Gelder, 2006; Jabbi et al., 2008; Pichon et al., 2009).

Precursors to the concept of vitality forms may be found in studies highlighting how movement style plays an important role in differentiating communicative gestures (Kendon, 1994), false vs. felt smiles (Ekman and Friesen, 1982), musical experiences (Clynes, 1973) and imitation or interactional synchrony during mother–infant interactions (Meltzoff and Gopnik, 1993; Trevarthen, 1998). This ability to perceive “how” an action is performed is an essential social skill, supporting implicit relational knowing (i.e., the ability to implicitly know how to behave with others, Stern, 1985; Lyons-Ruth et al., 1998), action imitation (Hobson and Lee, 1999; Hobson and Hobson, 2008), perception of individual differences based on movement (Loula et al., 2005) and adapting to moment-to-moment changes in social contexts (Thelen and Smith, 1994). Given their relevance in social interactions, it is important to analyze perception of vitality forms in children with autism spectrum disorders (ASD).

Autism spectrum disorders is characterized by deficits in social communication and social interaction and presence of repetitive and stereotyped behaviors (DSM-V, American Psychiatric Association [APA], 2013). However, children with ASD also show impairments in social skills related to perception of vitality forms, such as intuitive forms of social responsiveness (Travis et al., 2001), action imitation (see Vivanti and Hamilton, 2014, for a review), perception of biological motion and emotions based on movement (Blake et al., 2003; Nackaerts et al., 2012) and social motor synchrony (Fitzpatrick et al., 2016).

Neuroimaging studies have highlighted the existence of a tight link between the observation of others’ actions and their execution. In particular, studies on the human mirror neuron system (MNS) highlighted how parietal neurons involved in action planning have *mirroring* properties, i.e., these neurons selectively discharge both during the execution of an action and during the observation of the same action performed by another person (Rizzolatti and Craighero, 2004). Notwithstanding the existence of an ongoing debate on the function of the MNS in humans, which will probably be better understood only in due time with progress in neurophysiological findings, new techniques and parallel data from behavioral studies, various authors hypothesize that one of the functions of the MNS is to facilitate basic intersubjective understanding of others’ motor actions and action prediction (Rizzolatti and Sinigaglia, 2010). Given that MNS activation and action prediction are greater when observed movements fall within the observer’s own motor repertoires (Cross et al., 2006; Aglioti et al., 2008) some recent studies on motor atypicalities in individuals with ASD also suggest that these may impact motor resonance and understanding of others’ actions (see Cook, 2016 for a recent review). For example, Cattaneo et al. (2007) analyzed the link between action execution and action understanding in a group of seven children with ASD and eight TD controls aged 5 to 9 years. They asked children first to execute and later to observe two simple actions with different goals, but similar kinematics (i.e., picking up a piece of paper to put it in a container placed over the shoulder or picking up a piece of food to eat it). During both actions activation of the mouth-opening mylohyoid (MH) muscle was recorded. In the observation condition authors found pre-activation of the MH muscle during the reach-and-grasp phase only in the TD group. This pre-activation was mirrored in the observation condition once more only in the TD group, highlighting a link between proficiency in action execution and motor prediction of others’ actions during action observation (Cattaneo et al., 2007). Evidence from this study as well as from other studies on reach-to-grasp movements and motor action planning in children with ASD has led authors to hypothesize that motor problems may impact the ability to predict and make-sense of others’ actions in terms of motor intentions (i.e., “why”) in this population (Hughes, 1996; Fabbri-Destro et al., 2009; Forti et al., 2011; Sparaci et al., 2015). For example, specific difficulties in “why” understanding have been found in children with ASD compared to chronological age controls, mental age controls and children with other neurodevelopmental disorders affecting the social domain (i.e., Williams syndrome) (Boria et al., 2009; Sparaci et al., 2012, 2014).

In this view, hyper- and hypo-sensitivities to specific perceptual stimuli and differential movement patterns, often described within the etiology of ASD, influence how children with ASD perceive and make-sense of others’ behaviors (see De Jaegher, 2013 for a review). It is also interesting to note that within this approach it is possible to go beyond the individual child with ASD and toward a mayor focus on social interactions: while children with ASD may have difficulties in predicting others’ movements, their movements will result in turn difficult to interpret for others, leading to lack of fluidity in interpersonal sense-making (De Jaegher, 2013; Cook, 2016). In fact, given that vitality forms play an important role in making sense of other’s actions from birth onwards in children with TD, some authors suggest that early emerging disruptions in movement perception, motor timing and vitality dynamics may be at the basis of later emerging social impairments in children with ASD (Trevarthen and Delafield-Butt, 2013a,b). Therefore, it is surprising to find that to date only two studies have investigated perception of vitality forms in children with ASD in two very different experimental settings.

A first study by Hobson and Lee (1999) requested a group of 16 children and youths with ASD between 9 and 18 years of age and TD controls to imitate, after a delay, a series of observed actions with objects performed in different styles (e.g., taking a pipe rack with ridges and running a wooden stick across the ridges to make a vibrating sound in a rapid and forceful manner or more slowly and gently). These actions had a “what” (e.g., running the wooden stick across the ridges), a “why” (e.g., making a vibrating sound) and a “how” (e.g., the forceful or gentle manner of the action). While controls imitated all three aspects of the action, children with ASD were able to imitate the content of the movement (“what”) and its immediate goal (“why”), but rarely imitated its vitality form (“how”) (Hobson and Lee, 1999).

In a second study Rochat et al. (2013) asked 17 children and youths with ASD between 6 and 19 years of age and TD controls to observe videos of two actors performing different actions with or without objects (e.g., giving a mug, giving a high-five) executed with different vitality forms (e.g., vigorous or gentle). Videos were shown in pairs, some pairs differing in the type of action (i.e., “what” task) others in the vitality forms (i.e., “how” task), and participants were requested to judge whether videos differed or not. Results showed that performance of participants with ASD, compared to TD controls, significantly differed in the “how” task, while no differences emerged in the “what” task (Rochat et al., 2013).

Interestingly, both studies highlighted impairments in the perception of vitality forms in children and youths with ASD. However, these studies used procedures (i.e., imitation and similarity judgments), which involved a delay between stimulus presentation and participant responses, often enrolling procedural memory skills, which may be problematic for children with ASD (Gidley Larson and Mostofsky, 2008; Sparaci et al., 2015). They also included a wide age range considering both children and youths. The aim of the present study was to extend previous findings of impairments in the perception of vitality forms in children with ASD by using a task that did not require imitation nor similarity judgments, but rather an immediate evaluation of vitality forms. We also considered a narrower age range limiting our study to children in order to evaluate whether delays in the perception of vitality forms could also be captured at younger ages. Furthermore, we used a wider variety of vitality forms, to extend previous studies and understand whether difficulties with vitality forms in children with ASD could be linked to specific types or to a broad spectrum of vitality forms. Therefore, we requested a group of 15 children with ASD and a group of children with TD between 8 and 12 years of age to observe video-clips showing an action (i.e., move a bottle, a jar, or a can) performed with eight different execution times corresponding to different velocities and asked them to immediately judge verbally the vitality forms of the observed actions by using a five points Likert-type scale including the following judgments: “very rude,” “rude,” “so so,” “gentle,” “very gentle.”

Summing up, our main aims were: (1) to better evaluate perception of vitality forms in children with ASD using direct judgments; (2) to understand whether impairments could be narrowed down to specific types of vitality forms. In relation to these aims, our hypotheses were that we would find differences in vitality form perception in children with ASD relative to TD controls, in accordance with previous studies, but no predictions were made as to the second aim, as no previous data was available on this point.

## Materials and Methods

### Participants

Fifteen children with ASD and seventeen children with TD took part in the study. The study was approved by the Ethics Committee of the IRCCS Children’s Hospital Bambino Gesù, Rome (Protocol Number 486LB) and performed in accordance with the ethical standards laid down by the Declaration of Helsinki. The age of the group with ASD (14 males) ranged from 8 to 12 years (mean: 9.4 years; SD: 1.32). Children with ASD were recruited and evaluated at the “Bambino Gesù” Children’s Hospital, Rome (Italy). Children with ASD satisfied diagnostic criteria for ASD according to DSM-5. Documented diagnosis was provided by area clinicians, and confirmed using the Autism Diagnostic Observation Schedule (ADOS, Lord et al. 2000). According to ADOS scores, fourteen children in our group with ASD met criteria for autism and three children met the criteria for autism spectrum. Two children with ASD (autism diagnosis), during the training phase showed difficulties in paying attention to the task and were excluded from the sample (see below). Children in the TD group had a chronological age between 8 and 9 years (13 males; mean age: 8.8 years; SD: 0.39) and were recruited and evaluated at a public primary school in Rome, Italy. They were all primary speakers of Italian, had no previous history of language and/or learning disabilities, and no presence of ASD diagnosis in any immediate family member as documented by individual questionnaires completed by parents.

All children with ASD were administered the Weschler Intelligence Scale Children third edition (WISCIII; Wechsler, 1991) to evaluate verbal IQ and lexical comprehension. Children with TD were administered Raven’s Colored Progressive Matrices (RCPM, Raven et al., 1990) to assess non-verbal IQ and the Peabody Picture Vocabulary Test-Revised (PPVT-R; Dunn and Dunn, 1981) to evaluate lexical comprehension. ASD and TD groups displayed an IQ of 70 or above (see **Table 1**). In addition, all children with ASD and children with TD had good verbal comprehension skills as shown by the WISC III and PPVT-R, respectively (**Table 1**). Groups were matched for chronological and verbal age. Two samples *t*-tests showed no significant differences between groups in chronological age (*t*_32_ = -1.66, *p* > 0.05) and verbal age (*t*_30_ = 0.49, *p* > 0.05). All participants had normal or corrected to normal vision. The purpose of the study was presented and explained to teachers and educators as well as caregivers, the latter providing informed written consent.

**Table 1:** Groups characteristics including age, cognitive and diagnostic evaluation.

	Autism spectrum disorders	Typically developing controls
Sample size	15 (14 M, 1 F)	17 (13 M, 4 F)
Age	9.4 ± 1.3 (range 8–12)	8.8 ± 0.3 (range 8–9)
IQ total score	102.2 ± 12.4	115.9 ± 3.1
IQ verbal score	97.3 ± 18	100.1 ± 12.8
ADOS social interaction subscale	8.4 ± 2.2	n.a
ADOS stereotyped behaviors	2.8 ± 1.3	n.a
ADOS total score	11.2 ± 2.6	n.a

### Stimuli and Experimental Design

**FIGURE 1:**
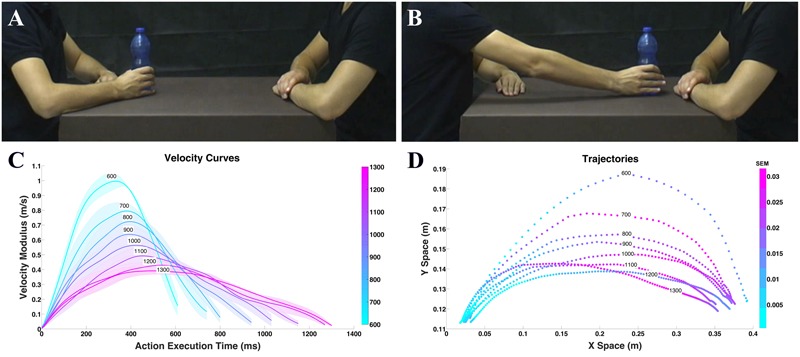
Example of video clips observed by the participants in experiment **(A,B)** and physical properties **(C,D)**. Frame representing the action performed with the bottle in the start position **(A)**; frame representing the same action in the end position **(B)**. Graph depicts mean velocity **(C)**, colored areas indicate the standard error of the mean (SEM). Graph depicts mean trajectory **(D)** profiles of the actions (i.e., move a bottle, a can, or a jar) performed by the actor with eight different execution times. Color intensity indicates the SEM.

Children were shown video-clips representing two actors, one of which moved one of three objects (i.e., a bottle, a can, or a jar) with his right hand toward the other actor (**Figures 1A,B**). The video stimuli were recorded using a high definition camera (Panasonic HCX 900) fixed at a 90° angle with respect to the actors (i.e., providing an allocentric point of view). VICON Motion Capture System (Vicon OMG, United Kingdom) with six infrared cameras (MX2 model, sampling frequency: 100 Hz.) was used to record the kinematic features of actor’s movements. In particular, six infrared cameras recorded the 3D position occupied by a marker placed on the back of each object and not visible in the video to avoid attracting children’s attention. In all videos, the actor performing the action started from the same initial position and reached the same final position (see also **Figures 1A,B** for a sample procedure). The three actions (i.e., moving a bottle, a can, or a jar) were performed with eight different execution times, which ranged from 600 to 1300 ms (i.e., 24 video stimuli in total). During action execution, the natural and ecological expression of vitality forms was preserved as much as possible avoiding excessive artificial manipulation of kinematic variables, changing only the execution time (i.e., velocity) while maintaining distance between the starting position and the ending position constant. Previous studies show that velocity profiles may play a key role in recognition of action style (Hidaka, 2012). Uniform distribution of stimuli duration was obtained by asking the actors to execute individual actions several times. Then, starting from the very rude stimulus (600 ms), stimuli differing 100 ms each other were selected. These stimuli were identical to the ones previously used in a behavioral and fMRI experiment conducted on healthy adults (Di Cesare et al., 2016). Note that, for all video-clips, the actors’ faces were not shown to avoid possible confound effects due to attention to facial expression. Each video lasted 2 s and was presented 10 times. The experiment included two sessions, each session consisted of 40 trials presented in a randomized order. Every experimental session lasted about 4 min while the whole experiment lasted about 8 min on average.

#### Physical Properties of Stimuli

During action execution, movements were characterized by different kinematics properties such as velocity, trajectory, and power. During stimuli recording, for each action we calculated the velocity and trajectory. After kinematic recording performed with the Vicon Nexus (OMG plc software, 100 Hz), data were analyzed using MATLAB (The Mathworks, Natick, MA, United States). The 24 stimuli (3 objects × 8 velocities) were compared pairwise for the purpose of verifying that trajectories differed by execution time while being similar by object. For this comparison we considered a metric related to the perceived distance (Turnwald et al., 2015): the Dynamic Time Warp (DTW; Berndt and Cliord, 1994; Ding et al., 2008). DTW allows measuring a distance between two time-series that have different duration by finding the correspondences between points in the time-series by means of a dynamic programming approach (see Supplementary Figure S1). This metrics was applied to the modulus of the 3D velocity vector of each trajectory resulting in a 24 by 24 symmetric matrix of pairwise distances (see Supplementary Figure S2). The distance matrix was analyzed to understand if, for every execution time (i.e., velocity), the distance among the objects inside each level of execution time (i.e., bottle 600 ms vs. jar 600 ms) was less than the ones of other execution times (i.e., bottle 600 ms vs. can 700 ms). Results of this analysis showed that for each execution time there was no major difference between the three objects considered. For this reason, we grouped the three objects together (i.e., bottle, can, jar) and calculated velocity average profiles (**Figure 1C**) and trajectories (**Figure 1D**).

### Paradigm and Task

Children were asked to sit at a table in front of a computer next to the experimenter. They were told that they would be watching a series of video-clips showing a person performing an action and that they would be asked to judge verbally, after each action, if the person performing the action was being gentle or rude. Judgments were made based on a five points Likert-type scale, including “very rude,” “rude,” “so so,” “gentle,” or “very gentle.” The choice of a five point Likert-type scale was supported by previous results obtained in a pilot study conducted on adults. In that study, participants were required to observe the same actions performed with different execution times and then to judge their vitality forms by using a continuous scale (score 0–100) with two poles (very gentle, score 0; very rude, score 100). Cluster analysis of this data highlighted that participants’ responses were organized in five different clusters. These results were used to build the five point Likert-type scale to be used with children and adult studies in order to simplify the task (Di Cesare et al., 2016). To verify semantic appropriateness of terms used in the Likert scale a pilot study was carried out on six TD children (mean chronological age: 6.5 years, range 6–7) previous to testing phase. All terms used proved appropriate for the selected age range. To avoid possible confound effects due to lack of attention, after the observation of each action, the children had the possibility to judge the action or to see it again. Prior to the experimental session, children underwent a training session, with similar stimuli to those used during the experiment, to familiarize with the task and the Likert-type scale. In the first part of this training, using E-Prime software (Psychology Software Tools, Inc., United States), stimuli were presented and children were asked to observe the actions. In the second part of the training, children observed the same actions and, using the Likert-type scale, judged their vitality forms. Only children that successfully completed the training session were included in the final sample. Two children with ASD, showed difficulties in paying attention to the video-clips during the training session and therefore did not carry out the experimental session, but were offered an alternative activity and were excluded from the final sample. All statistical analyses were carried out using R 3.2.2 (R development Core Team) packages: lme4 – Linear Mixed-Effects Models using Eigen and S4 and lmer Test for Mixed Models analysis; g models for frequency models analysis.

## Results

### Frequency Response Analysis

**FIGURE 2:**
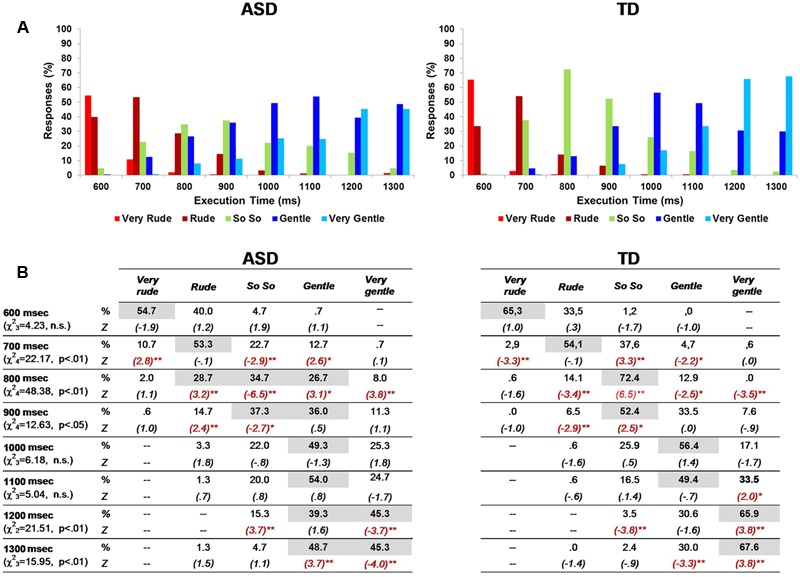
Percentage of responses in ASD and TD groups **(A,B)**. In each category response, the most frequent percentages are highlighted in gray color. Asterisks indicate significant adjusted standardized cell residuals (^∗^*p* < 0.05; ^∗∗^*p* < 0.01), pointing out the different response categories between ASD and TD children in each action execution time **(B)**.

Merging numerical scores into a single group mean allowed to conceal some response trends and subtle differences between groups. Thus, we verified the presence of differences between groups in single category response frequencies for each execution time using a two-way chi square test (**Figure 2**) and direction in which it occurred (i.e., adjusted standardized cell residuals). At T_600__ms_, the response categories used by children with ASD and controls were not statistically different: “very rude” and “rude” responses prevailed in both groups, with similar percentages. At T_700__ms_, the “rude” response was the most frequently chosen one by both groups. However, children with TD used the intermediate category “so so” significantly more often than children with ASD (standardized adjusted cell residuals: *Z* = 3.3, *p* < 0.01), who tended to continued evaluating the action as “very rude” (*Z* = 2.8, *p* < 0.01). At T_800ms_, this tendency appeared even greater: while the great majority of children with TD (72.4%; *Z* = 6.5, *p* < 0.01) defined the observed action as neutral (i.e., “so so”), children with ASD produced responses that were quite homogenously distributed among “rude,” that was still more common in this group (28.7%; *Z* = 3.2, *p* < 0.01), “so so” (significantly less recurrent than in TDs: 34.7%; *Z* = -6.5, *p* < 0.01) and “gentle” (more frequent than in TD: 26.7%; *Z* = 3.1, *p* < 0.01). At T_900ms_, children with ASD shared their preference between “so so” (37.3%, *Z* = -2.7) and “gentle” (36%, *Z* = 0.5); even at this execution time, the percentage of “rude” responses was greater than in the TD group (14.7% versus 6.5%, *Z* = 2.4, *p* < 0.05), who provided mostly “so so” (52.4%, *Z* = 2.5) and “gentle” (33.5%) judgments. At T_1000ms_ and T_1100ms_, both groups showed similar response distributions, preferring the “gentle” category, even if at T_1100ms_ the “very gentle” extreme was more frequent among controls (33.5% versus 24.7%; *Z* = 2.0). Finally, responses at T_1200ms_ and at T_1300ms_ differed between groups: children with TD stated more frequently that the action was “very gentle” (respectively: 65.9% versus 45.3%, *Z* = 3.8; 67.6% versus 45.3%, *Z* = 3.8), whereas children with ASD equally divided their responses between “gentle” and “very gentle.”

### Differences with Respect to Expected Responses

**FIGURE 3:**
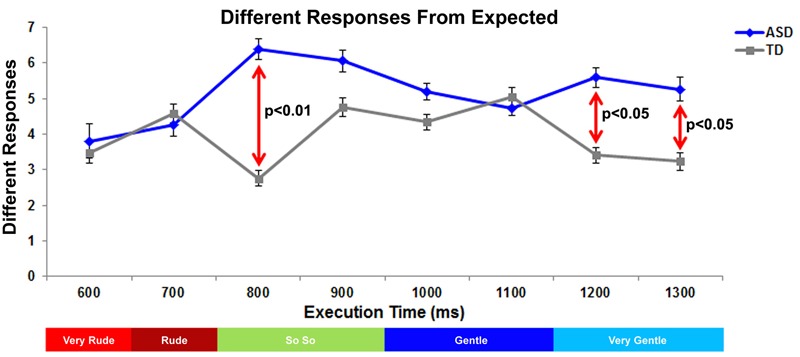
Graph depicts the number of responses of vitality forms differing from expected judgment in ASD and TD groups. The red lines indicate the significant differences between ASD and TD groups. Below the graph, the colored bar indicates the expected response for each action execution time.

For each stimulus the significantly prevalent category (one-way chi square test) in the TD group was considered as the “expected response” and used as normative standard: “very rude” at T_600ms_, “rude” at T_700ms_, “so so” at T_800ms_ and T_900ms_, “gentle” at T_1000ms_ and T_1100ms_, “very gentle” at T_1200ms_ and T_1300ms_ (see also **Table 2**). Consequently, all judgments differing from the expected response were analyzed. Different responses were entered in a Generalized Linear Mixed–effect Models Analysis (GLMM) considering the random effects of the action execution time nested within the subject identity as random effect, using the forward stepwise method described in Frequency Response Analysis. Results showed a significant effect of Group (*F*_[1;240]_= 18.71, *p* < 0.01) and a significant interaction between Execution Time and Group (*F*_[7;224.76]_= 2.99, *p* < 0.05). Children with ASD differed in vitality forms judgments from children with TD in almost all phases (**Figure 3**). In particular, significant differences between groups were present at T_800ms_ (*p* < 0.01; Bonferroni planned contrasts), T_1200ms_ (*p* < 0.05) and T_1300ms_ (*p* < 0.05).

**Table 2:** Judgments categories in TD group.

	600 ms		700 ms		800 ms		900 ms		1000 ms		1100 ms		1200 ms		1300 ms	
	*N*	Residue	*N*	Residue	*N*	Residue	*N*	Residue	*N*	Residue	*N*	Residue	*N*	Residue	*N*	Residue
Very Rude	**111**	**54.3**	5	-29.0	1	-41.5	–	–	–	–	–	–	–	–	–	–
Rude	57	0.3	**92**	**58.0**	24	-18.5	11	–31.5	1	-41.5	1	-41.5	–	–	–	–
So So	2	-54.7	64	30.0	**123**	**80.5**	**89**	**46.5**	44	1.5	28	-14.5	6	-50.7	4	-52.7
Gentle	–	–	8	-26.0	22	–20.5	57	14.5	**96**	**53.5**	**84**	**41.5**	52	-4.7	51	-5.7
Very Gentle	–	–	1	-33.0	–	–	13	–29.5	29	-13.5	57	14.5	**112**	**55.3**	**115**	**58.3**
Chi-squared	104.835		202.059		210.941		99.647		112.212		90.941		99.718		109.565	
	*p* < 0.01		*p* < 0.01		*p* < 0.01		*p* < 0.01		*p* < 0.01		*p* < 0.01		*p* < 0.01		*p* < 0.01	

*The expected response for each action execution time is highlighted in bold.*

### Discrimination Ability

**FIGURE 4:**
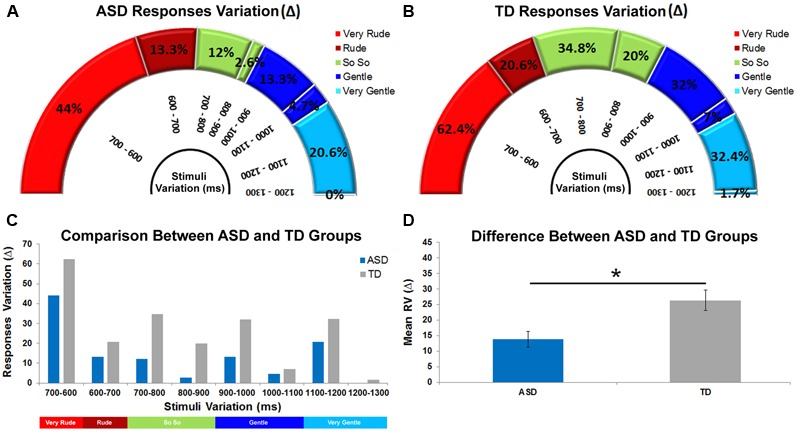
Delta values relative to response categories obtained for ASD and TD groups in eight different stimuli comparisons **(A,B)**. High Δ values denote greater ability to discriminate two contiguous stimuli while low Δ values indicate a lower discrimination ability. Discrimination ability comparison between ASD and TD groups **(C)**. Difference between ASD and TD groups relative to the mean discrimination ability **(D)**. The bars indicate the SEM. Asterisk indicates significant difference between ASD and TD groups (*p* < 0.005).

In the present study, the eight experimental stimuli differed one from the other by a constant time interval of 100 ms. To assess how ASD and TD groups adapted their responses to stimulus variation (i.e., discrimination ability), we considered the variation of correct responses for each of the eight stimuli intervals (Very Rude: 700–600; Rude: 600–700; So So: 700–800, 800–900; Gentle: 900–1000, 1000–1100; Very Gentle: 1100–1200, 1200–1300). This was done by calculating differences in Delta (Δ) (i.e., the difference in correct responses expressed as an absolute value) provided by ASD and TD groups during observation of each stimulus, compared with correct responses provided during the observation of the previous stimulus. For example, considering the *very rude* category and the stimulus interval comprised between T_700ms_ and T_600ms_, we calculated a Delta value of 44% for the ASD group [(10.7%)_T700__ms_ – (54.7%)_T600__ms_; **Figure 4A**] and of 62.4% for the TD group [(2.9%)_T700ms_ – (65.3%)_T600__ms_; **Figure 4B**]. A high Delta value indicated that physical difference in a specific stimulus interval was well perceived, while low Delta value indicated that this difference was not well perceived (i.e., 1200–1300). Delta values were calculated for all vitality form categories (Very Rude: Δ_700-600_; Rude: Δ_600-700_; So So: Δ_700-800_, Δ_800-900_; Gentle: Δ_900-1000_, Δ_1000-1100_; Very Gentle: Δ_1100-1200_, Δ_1200-1300_; see **Figure 4C**). After the evaluation of all Delta values, we calculated the *mean Δ value* (ASD:13.8% SEM = ± 4.9; TD:26.3% SEM = ± 6.6) and carried out an independent samples *t*-test that revealed a significant difference (*p* < 0.005) between ASD and TD groups (**Figure 4D**).

## Discussion

During social interactions, perception of vitality forms allows to capture “how” an action is performed. The concept of vitality forms is meant to capture the style of an action, rather than its content (i.e., “what” is being done) or the motor intention characterizing it (i.e., “why” it is being done) (Stern, 2010). Vitality forms are distinct from emotions, but play an important role in everyday social interactions and their importance has been highlighted throughout the lifespan, from infancy to adulthood (Brazelton et al., 1975; Trevarthen, 2006; Stern, 2010). While many studies have been dedicated to emotion perception in children with ASD (Sinigaglia and Sparaci, 2010; Uljarevic and Hamilton, 2013), only two studies to date have addressed perception of vitality forms in children and youths with ASD. The first aim of the present study was to extend previous studies assessing whether vitality forms expressed during a simple social interaction were immediately perceived by children with ASD between 8 and 12 years of age; the second aim was to better understand whether possible differences emerging in vitality forms perception in children with ASD could be narrowed down to specific *types* of vitality forms.

Data showed that, children with ASD differed from children with TD in the perception of vitality forms. This result extends previous findings by Hobson and Lee (1999) and by Rochat et al. (2013) who found similar differences, respectively, in the execution and the observation of vitality forms, using different methodologies (i.e., imitation and similarity judgments), which involved longer delays between stimulus presentation and assessment, and considered broader age ranges. The present results allow us to state that perception of vitality forms in ASD is already impaired in childhood. Furthermore, by allowing for a shorter delay between stimulus presentation and perception of vitality form assessments we were able to better exclude possible interference of other skills (e.g., procedural memory) often impaired in ASD.

Interestingly, children with ASD recognized “very rude,” “rude,” and “gentle” vitality forms, while they showed difficulties in recognizing “neutral” and “very gentle” vitality forms. An important aspect highlighted by these data was that, observing different vitality forms, children with ASD recognized positive (i.e., “gentle”) and negative (i.e., “very rude,” “rude”) vitality forms while they had difficulties in recognizing neutral vitality forms (i.e., “so so”). These results extend previous findings by providing data on the perception of specific *types* of vitality forms in children with ASD. Our findings suggest that ASD children mostly detect extreme expressions of vitality, while lacking a more nuanced perception of vitality forms that often characterizes everyday actions. Indeed, in everyday life most actions and in particular those with objects are performed with more nuanced vitality forms rather than extreme ones, which we resort to only in extreme situations.

It must, however, be considered that in real-life social contexts, besides hand and arm movements, other important information is present, such as facial expression/the gaze (visual information), voice tone (acoustic information), or body contact (tactile information). This information, which may facilitate or enhance perception of vitality forms, was absent in our study to simplify study design and avoid possible distractions. This is one of the limits of the present study, which may be overcome in the future by considering different types of stimuli while assessing vitality form perception in children with ASD. In fact, it may be plausible that visual information is not sufficient for children with ASD to encode vitality forms correctly and that the use of alternative perceptual information may help vitality forms perception. A second limit of the present study is the use of an allocentric point of view in stimulus recording in order to maximize the perception of motion and the velocity profile of performed actions. Possibly, using of an egocentric point of view may lead to different results due to changes in stimuli salience. However, evaluation of changes in vitality forms due to allocentric vs. egocentric point of view have not been documented in the literature and need to be addressed in further studies.

Another important result of the present study is that children with ASD differed from TD controls in their ability to discriminate differences between stimuli. More specifically, varying stimuli presentation, children with ASD showed reduced variation in responses with respect to controls. This result underscores difficulties in children with ASD in perceiving differences between two contiguous stimuli (100 ms) and seems to indicate that, during action observation, children with ASD need greater stimuli variations than children with TD to detect their differences in terms of vitality forms (smallest change detected in ASD > 100 ms). These findings are in line with recent results by Fitzpatrick et al. (2016) showing that adolescents with ASD have difficulties in synchronizing their movements with others’ movements during both spontaneous and intentional interpersonal coordination. In fact, lack of sensitivity to variation in vitality forms highlighted in our study may have an impact on motor synchrony during social interactions: if changes are not perceived it is difficult to adapt to them. These difficulties in perceiving differences in others’ actions may be due to motor atypicalities in individuals with ASD given the link described in the Introduction between observation and execution of motor actions. For example, Cook et al. (2013) observe that in adults with ASD the ability to recognize if an observed human movement is ‘natural’ is correlated to the individual’s ability to appropriately execute the kinematics of the same movement (i.e., individuals with ASD that were able to perform an arm waving movement with velocity, acceleration and jerk similar to the one observed in TD controls, were also better at distinguishing among a set of motion-morphed stimuli of a hand performing waving movements the more human-like ones). This study on adults, alongside the study on children conducted by Cattaneo et al. (2007) described above, suggest that the flip side of investigating vitality forms recognition in ASD may be in analyzing vitality form execution. Interestingly, Hobson and Lee (1998) investigated vitality execution during greeting and farewell behavior in children with ASD compared to the same behaviors in children with learning difficulties. Results showed that children with autism performed fewer greeting and farewell behaviors, but their social interactions were also judged as manifesting less engagement (Hobson and Lee, 1998). Therefore, future studies may attempt to investigate vitality forms both during the observation and during the execution of similar actions to fully understand social coordination in children with ASD and its link with motor functions (Bhat et al., 2011).

Functional magnetic resonance imaging studies conducted on adults have recently allowed to highlight neural correlates involved in vitality forms processing. More specifically, Di Cesare et al. (2016) using the same stimuli employed in the present study showed that observation of “rude,” “neutral,” and “gentle” vitality forms relative to controls produced activation of the dorso-central insula. Authors also found that the same area is also involved in the expression of action vitality forms (Di Cesare et al., 2015, 2017). Pooling together, these findings strongly suggest the existence of a mirror mechanism for action vitality forms in the dorso-central insula. Unlike the mirror mechanism located in the parietal and frontal areas (Rizzolatti et al., 2014; Rizzolatti and Sinigaglia, 2016), which plays a role in action goal understanding, the action mirror mechanism located in the insula allows expressing our own vitality forms and to understand vitality forms expressed by others. On the basis of neurophysiological findings it is plausible to hypothesize that impairments observed in children with ASD during vitality forms recognition could be ascribed to an incorrect functioning of the dorso-central insula or of the cortical areas functionally connected with it, such as the inferior parietal lobe and the inferior frontal gyrus. In line with this hypothesis various studies have reported structural and functional alterations in the insula in individuals with ASD. In particular, alterations of gray matter volume in the insula have been reported in individuals with ASD (Kosaka et al., 2010; Cauda et al., 2011; Ecker et al., 2012). Furthermore, a meta-analysis by Di Martino et al. (2009) reported a hypo-activation of the anterior insula in individuals with ASD relative to individuals with TD during the execution of different social tasks. Finally, regarding brain connectivity, previous resting-state fMRI studies conducted on individuals with ASD showed reduced functional connectivity of the insula with other brain regions including the amygdala and the somatosensory cortex (Ebisch et al., 2011; Di Martino et al., 2014). However, most studies so far focused on the role of the insula on emotion understanding in ASD and further neurophysiological studies specifically dedicated to perception of vitality forms in ASD are needed to confirm the existence of a link between differences in vitality forms perception and functioning of the dorso-central insula.

## Conclusion

Our findings allow extending previous data on impairments in vitality forms perception in ASD, while shedding new light on how specific types of vitality forms as well as changes in vitality forms are particularly difficult to perceive for children with ASD. Taken together these finding may have implications for professionals and caregivers interacting with children with ASD, who may attempt to facilitate perception of vitality forms by relying less on subtler types of vitality forms and considering difficulties in change perception. While a subtle expression of vitality may be easily readable for a child with TD, a child with ASD may not perceive it. This may lead to lack of intersubjective coordination and understanding. Knowing this, professionals and caregivers may try to attract the child’s attention to specific vitality forms that are harder to perceive and allow for more time to be dedicated to the development of this skill. Available data on vitality forms perception in children with ASD suggest that this is a relevant skill that we need to acquire in order to obtain that fluidity of interaction that characterizes our everyday encounters with others.

## Author Contributions

Designed the research: GDC, LM, AP, LS, DM, and SV. Performed the research: GDC, LM, LS, and GG. Analyzed the data: GDC, AP, and ER. Wrote the paper: GDC, LS, and AP.

## Conflict of Interest Statement

The authors declare that the research was conducted in the absence of any commercial or financial relationships that could be construed as a potential conflict of interest.
