# Supplementary material for: Differences in Action Style Recognition in Children with Autism Spectrum Disorders
Source: Front Psychol. 2017 Sep 4;8:1456. doi: 10.3389/fpsyg.2017.01456 (PMC5591610; doi:10.3389/fpsyg.2017.01456)
Supplement: Supplementary file 1 [file Data_Sheet_1.DOCX]

**Supplementary Materials**

**Materials and Methods**

**2.2.1 Physical properties of stimuli**

This section contains additional information that is useful for better understanding the nature of the trajectories employed for the stimuli, and, in particular the role of the Dynamic Warp Transform (DTW) as a distance metric that allows for considering equivalent the trajectories with same execution time but different object.


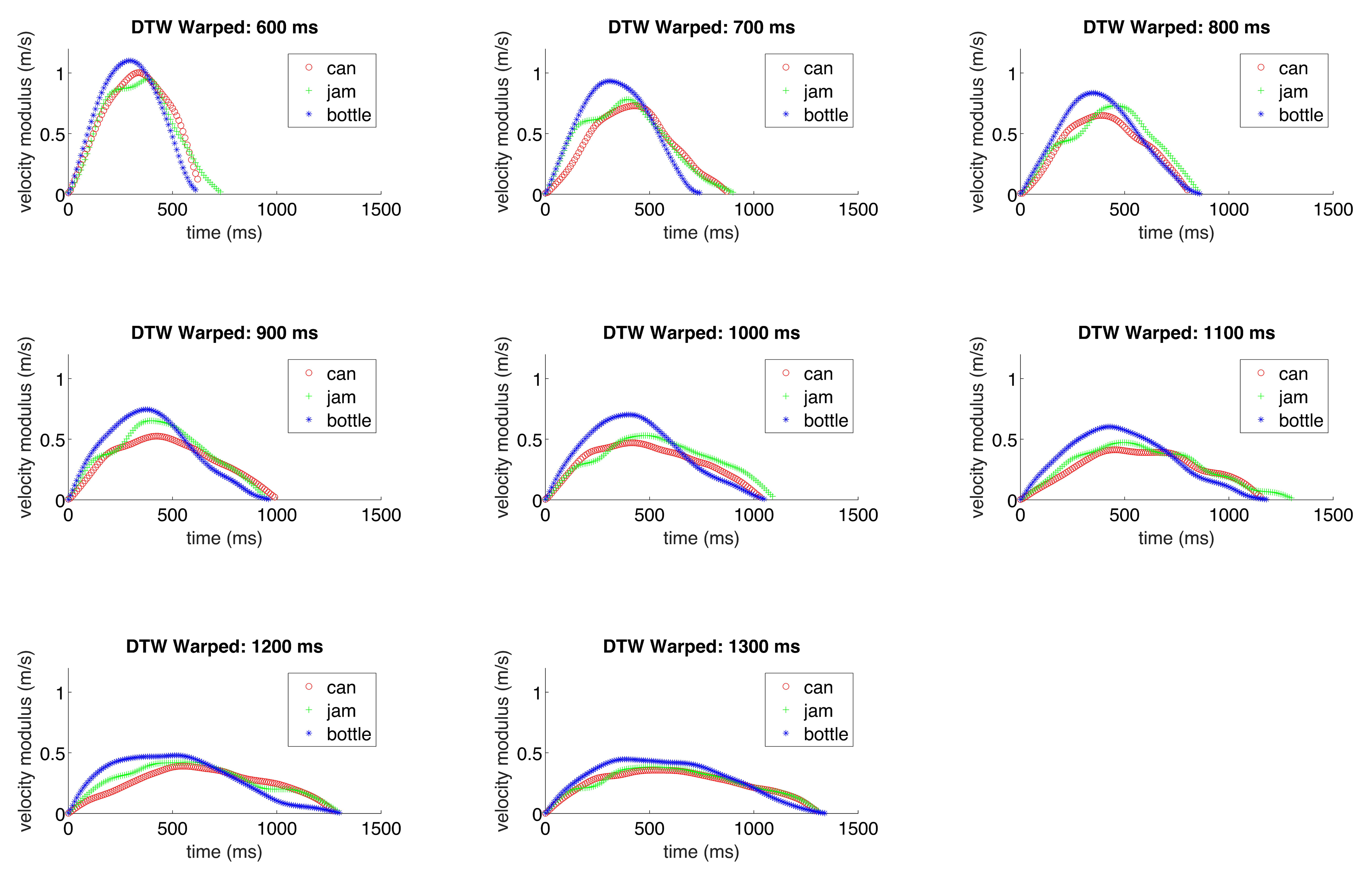


**Figure S1**: This figure shows, for each execution time, the transformation of the DTW technique for warping the velocity curves. For this plot each jam and bottle curve is transformed against the can of the same execution time. The DTW distance is the Euclidean distance between pair of curves after warping.


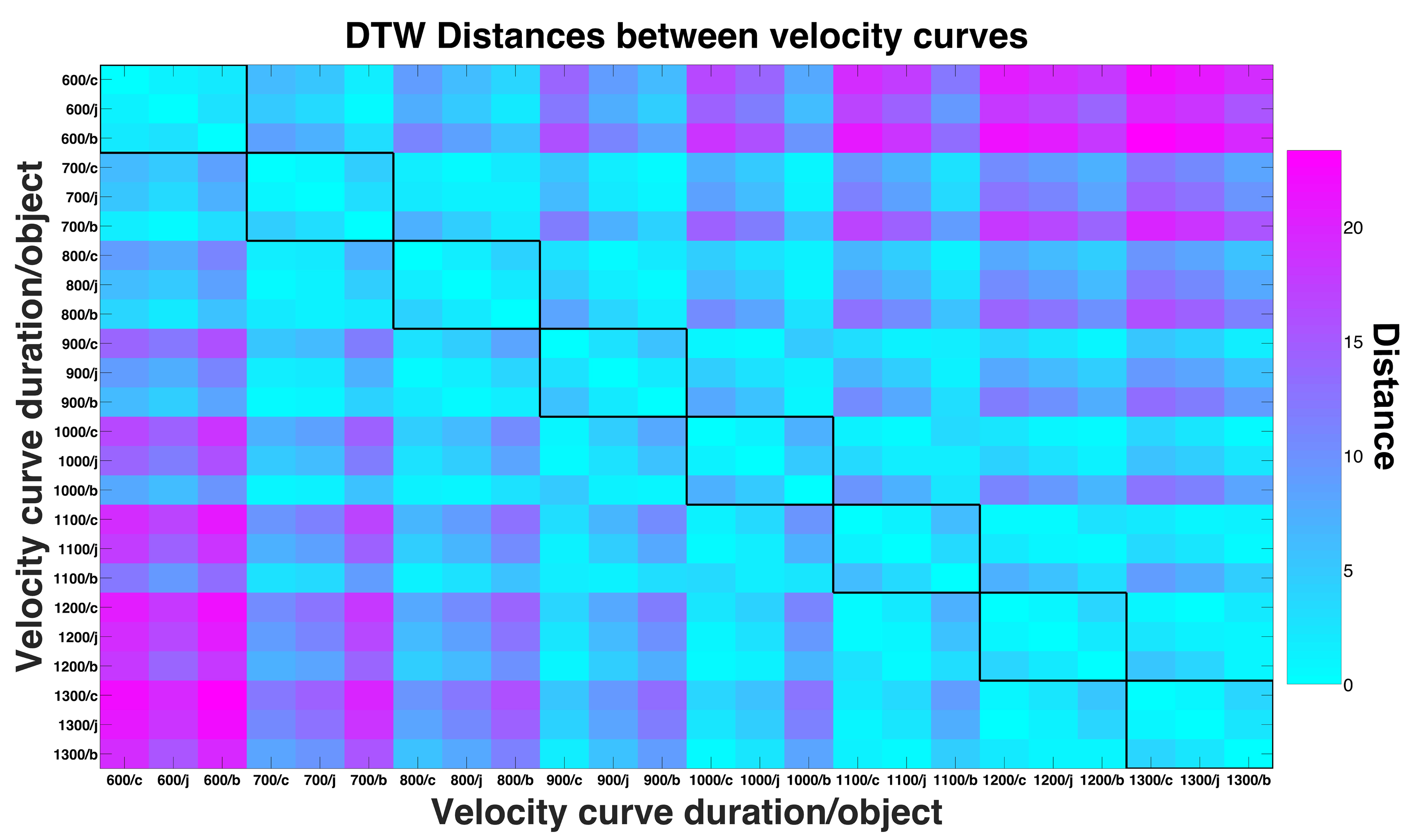


**Figure S2**: This figure shows the pairwise symmetric matrix of DTW distances between each velocity curve by execution time and object (b, bottle; j, jam; c, can). Black rectangle highlights the curves belonging to the same execution time. The distance is shown using colors with a scale ranging from light blue to purple. More specifically, for each execution time, small DTW distances among curves are highlighted with light blue color, while larger distances are shown in purple color.

**Results**

GLM Results

Participants’ vitality forms judgments were automatically converted by E-Prime software in numerical scores (very rude=5; rude=4; so so=3; gentle=2; very gentle=1). Measures were entered in a General Linear Mixed–effect Models analysis (GLMM), considering the effects of the action execution time (8 levels) nested within the subject identity as random effect. A residual funnel graph confirmed the homoscedasticity of the models residuals; Shapiro tests established that they did not significantly diverge from normality. Models were set up with a forward stepwise procedure: starting from a null random model, predictors (principal effects: Execution time and Group; then the interaction term) were added, verifying if they determined a reliable change into the model fitting (Akaike Information Criterion – AIC – and Schwarz’s Bayesian Information Criterion – BIC). The results of the GLM analysis indicated a significant, strong effect of the action execution time on the judgments (F_[7;223.3]_=406.03, p<0.01), but no significant differences between group appeared, and the Time by Group was only near significance (F_[7;223.3]_=1.9, p=0.07). Planned contrasts with Bonferroni correction were used for paired comparisons: ASDs’ scores were significantly greater only at T_1200_ and T_1300_ (p<0.05) and quite near significance at T_1100_ (p=0.08).
